# Small Molecule Binding, Docking, and Characterization of the Interaction between Pth1 and Peptidyl-tRNA

**DOI:** 10.3390/ijms141122741

**Published:** 2013-11-19

**Authors:** Mary C. Hames, Hana McFeeters, W. Blake Holloway, Christopher B. Stanley, Volker S. Urban, Robert L. McFeeters

**Affiliations:** 1 Department of Chemistry, University of Alabama in Huntsville, 301 Sparkman Drive, Huntsville, AL 35899, USA; E-Mails: mcg0001@uah.edu (M.C.H.); hk0003@uah.edu (H.M.); beb0004@uah.edu (W.B.H.); 2 Oak Ridge National Laboratory, Biology and Soft Matter Division, P.O. Box 2008, Oak Ridge, TN 37831, USA; E-Mails: stanleycb@ornl.gov (C.B.S.); urbanvs@ornl.gov (V.S.U.)

**Keywords:** peptidyl-tRNA hydrolase, small angle neutron scattering, enzyme-substrate complex, docking, inhibition

## Abstract

Bacterial Pth1 is essential for viability. Pth1 cleaves the ester bond between the peptide and nucleotide of peptidyl-tRNA generated from aborted translation, expression of mini-genes, and short ORFs. We have determined the shape of the Pth1:peptidyl-tRNA complex using small angle neutron scattering. Binding of piperonylpiperazine, a small molecule constituent of a combinatorial synthetic library common to most compounds with inhibitory activity, was mapped to Pth1 via NMR spectroscopy. We also report computational docking results, modeling piperonylpiperazine binding based on chemical shift perturbation mapping. Overall these studies promote Pth1 as a novel antibiotic target, contribute to understanding how Pth1 interacts with its substrate, advance the current model for cleavage, and demonstrate feasibility of small molecule inhibition.

## 1. Introduction

Removal of bound peptides from peptidyl-tRNA is essential for cell viability in all domains of life. Peptidyl-tRNAs are generated when ribosomes abort translation prematurely [1–3], which occurs on average 10% of the time [4]. Peptidyl-tRNAs are released by ribosome recycling factor and elongation factor-G [4,5] or fall-off at a rate depending on the attached tRNA [6]. Accumulation of peptidyl-tRNAs also results from the expression of minigenes or short ORFs [7–9]. To avoid excessive build-up of peptidyl-tRNAs and resulting tRNA starvation leading to rapid cell death, it is vital for cells to maintain peptidyl-tRNA hydrolase (Pth) activity.

Although Pth activity is universal, the highly conserved Pth1 enzyme in bacteria differs greatly from the multiple Pth systems found in eukaryotes. While essential in bacteria, loss of Pth1 function does not alter yeast viability [10]. Further, no sequence or structural homology exists between Pth1 and other eukaryotic Pth enzymes and their cleavage mechanisms are unrelated [11–14]. Thus the essential function, high conservation across bacterial species, and apparent lack of an essential human equivalent make Pth1 a much needed new target for antibacterial development.

Structures of 21 kDa monomeric Pth1 have been solved for several bacterial species [15–19]. As predicted from the high degree of amino acid sequence similarity, all have nearly identical backbone folds. Pth1 family members are globular, single domain proteins that have a central mixed β-sheet surrounded by α-helices. Insight into substrate binding and recognition comes from studies of mini-substrates and a crystal structure of Pth1 in complex with a tRNA CCA-acceptor TΨC domain [20–22]. Two proximal binding sites for small molecule inhibitors, one on each side of the peptide binding channel surrounding the catalytically essential residue His20 (as numbered in *E. coli* Pth1), were suggested by molecular modeling [15].

The identification of Pth1 inhibitory activity in natural product extracts [23,24] and commonality of extracts that inhibit Pth1 from multiple bacterial species solidifies this assertion and further supports the possibility of broad spectrum inhibition. However, the structure of the peptidyl-tRNA bound complex, molecular mechanism of the reaction, and potential for small molecule inhibition remains unclear.

Herein we report the first overall shape determination of the Pth1:peptidyl-tRNA complex using small angle neutron scattering (SANS). We also demonstrate specific binding of a small molecule and characterize the interaction interface. Computational analysis indicates important interactions and potential for improvements. This work represents the first small molecule binding to Pth1, providing the foundation for continued structure based drug design.

## 2. Results

### 2.1. Small Angle Neutron Scattering

**Figure 1:**
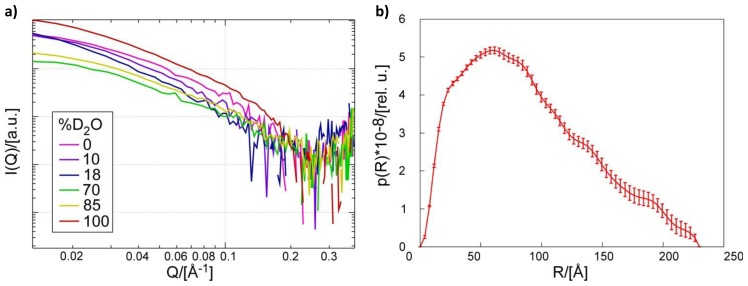
Small Angle Neutron Scattering. (**a**) Scattering curves for Pth1H20R:peptidyl-tRNA complex from contrast series measurements taken at buffer D_2_O concentrations of 0%, 10%, 18%, 70%, 85%, and 100%; (**b**) Pairwise distance distribution function of scattering data from complex in 100% D_2_O generated in GNOM [25].

SANS data were collected from samples of catalytically inactive Pth1H20R:peptidyl-tRNA complex in buffer at six different H_2_O:D_2_O ratios, Figure 1a. The average radius of gyration, *R*_g_, was 63 ± 4 Å from Guinier analysis of the 100% D_2_O sample, in agreement with dynamic light scattering estimates of 65 ± 7 Å. For illustration, the distribution of distance pairs resulting from SANS data collected at 100% D_2_O is shown in Figure 1b. The maximum dimension, *D*_max_, of the Pth1:peptidyl-tRNA complex was 230 Å, which was used as an upper limit for the MONSA modeling. Structural parameters *R*_g_ and *D*_max_ were consistent for all measurements.

### 2.2. Shape of the Pth1:peptidyl-tRNA Complex and Their Relative Orientation

**Figure 2:**
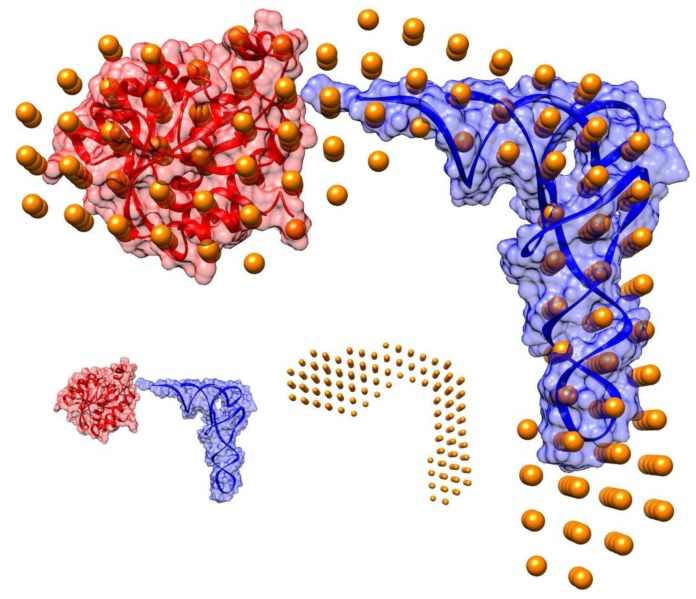
Model of Pth1:peptidyl-tRNA Complex. The overall shape of the Pth1H20R:peptidyl-tRNA complex is shown in gold spheres. *E. coli* Pth1 (PDBID: 2PTH) and tRNA^Phe^ (PDBID:1EHZ) were fit into the mass density. Pictured in the inset (lower right) are the individual components: tRNA^Phe^ in blue, Pth1 in red, and the calculated shape in gold spheres.

Using the *R*_g_ value as an upper limit on the size of the search space, the overall shape of the Pth1H20R:peptidyl-tRNA complex was solved. Modeling results are shown in Figure 2 with atomic coordinates from *E. coli* Pth1 (PDBID: 2PTH) and tRNA^Phe^ (PDBID: 1EHZ) modeled in. The shape of the envelope of the complex suggests the location of the tRNA portion of the substrate and that of Pth1. Using available information on the location of the active site residues [26,27] and the proposed peptide binding channel [16] for Pth1 with the structure of the enzyme:TΨC loop complex [22], Pth1 and tRNA were successfully modeled into SANS envelope. The high resolution coordinates of *E. coli* Pth1 (2PTH.pdb) were fitted into the low resolution SANS model restricting the search to the part of the model that was not filled by the tRNA density using SUPCOMB. The normalized spatial discrepancy (NSD) value determined by SUPCOMB was 0.54, indicating a good fit between the two volumes (*i.e.*, NSD below 1.0) [28]. In the resulting structure, Pth1 was oriented such that the positive patch and catalytic His20 residue were near the tRNA 3′ terminus. The high heterogeneity of the substrate resulted in a shape reflecting the various peptidyl-tRNA species and therefore, fitting the tRNA portion in the bead model has not been as straight forward as that of Pth1. In the end, the rigid tRNA^Phe^ crystal structure was positioned manually leaving some unaccounted volume in the anticodon region. Variation in this region comes from plasticity of the tRNA molecule as a whole [29], mobility in the anticodon region [30], and heterogeneity of the peptidyl-tRNA used for data collection.

### 2.3. Piperonylpiperazine Binding and Interaction with Pth1

**Figure 3:**
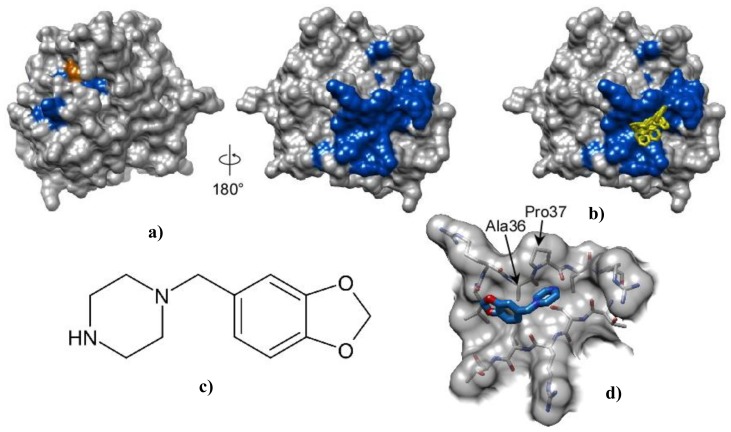
Interaction and docking of *E. coli* Pth1 with piperonylpiperazine. (**a**) Surface representation of *E. coli* Pth1 (PDBID:2PTH) shown with catalytically important His20 in orange. From NMR data, residues with ^1^H–^15^N resonances affected by interaction with piperonylpiperazine are in blue; (**b**) Docking: The six lowest energy orientations of piperonylpiperazine are shown in yellow; (**c**) Structure of piperonylpiperazine; (**d**) An enlarged view of the piperonylpiperazine binding site.

From screening of a synthetic library of compounds for inhibitory activity against Pth1, we have found piperonylpiperazine is one of the prevailing common constituents of inhibitory compounds. The binding of piperonylpiperazine to wild type *E. coli* Pth1 was studied by NMR spectroscopy. Binding affinity was relatively low, with complete saturation not observable at a molar ratio of 64:1 (piperonylpiperazine:Pth1). Fast exchange on the NMR time scale was observed from migration of resonances to their bound positions. Piperonylpiperazine did not inhibit Pth1 activity and did not directly interact with the peptide binding site of the substrate, instead binding to the opposite side of the molecule, Figure 3.

To further investigate the interaction of piperonylpiperazine with Pth1, molecular docking was pursued. The docking search space for piperonylpiperazine binding to Pth1 was centered on the Pth1 face indicated from NMR chemical shift perturbation mapping. Piperonylpiperazine was found to bind in a shallow depression with a calculated binding energy ranging from −3.8 and −4.4 kcal/mol. Significant interaction with the hydrophobic residues (Ala36–Pro37–Leu38) leading up to the edge of the central mixed β-sheet were observed in all poses. Figure 3b shows the six lowest energy poses out of 36 calculated.

In bacterial culture, millimolar concentrations of piperonylpiperazine did not inhibit *E. coli* growth and no inhibition of Pth1 cleavage was observed from an *in vitro* activity assay [23,24] for concentrations exceeding 10 mM piperonylpiperazine. Thus, even though piperonylpiperazine was a common constituent of Pth1 inhibitors, it does not itself inhibit Pth1 function. Rather, it seems that the interaction with Pth1 makes piperonylpiperazine a suitable anchor for the other constituents of Pth1 inhibitors.

## 3. Experimental Section

### 3.1. Expression and Purification of *E. coli* Pth1

Wild-type and catalytically inactive H20R Pth1 from *E. coli* were expressed in W3110 *E. coli*. Cells were grown in minimal M9 media at 37 °C to an OD_600_ of 0.7, at which point the temperature was dropped to 30 °C and protein production in the culture was induced with 1 mM isopropyl β-d-1-thiogalactopyranoside (IPTG). Pth1 was expressed for approximately 6 h before the cells were harvested by centrifugation. Expression and solubility were verified by SDS-PAGE. Purification of Pth1 was performed as previously described [23]. Briefly, pelleted cells from Pth1 were resuspended in lysis buffer containing 50 mM NaHPO_4_, 300 mM NaCl, and 2 mM DTT, pH 7.4. Fifteen milligrams of lysozyme was added and the lysate was allowed to sit at room temperature for 30 min before centrifugation at 18,000 rpm for 30 min at 4 °C. The supernatant was loaded onto a His-Trap FF column equilibrated with lysis buffer and eluted with 150 mM imidazole. Pooled fractions were dialyzed in 20 mM Bis–Tris, 50 mM NaCl, and 2 mM DTT and concentrated to ~2 mM.

### 3.2. Production of Bulk Peptidyl-tRNAs

Using a bacterial strain with temperature sensitive Pth1 [31,32], bulk peptidyl-tRNA was produced using a modification of previously reported protocol [33]. C600 Pth(Ts) was grown in LB at 30 °C to an OD_600_ of 0.4. The temperature was then shifted to a non-permissive 42 °C for 1 h. Cells were harvested by centrifugation and frozen. Cell pellets were resuspended in cold 0.3 M NaOAc, 10 mM EDTA, pH 4.5, followed by phenol/chloroform extraction. Peptidyl-tRNA was precipitated by adding 2.5 volumes of cold ethanol to the aqueous fraction. After pelleting by centrifugation, the pellet was washed twice with ethanol. Peptidyl-tRNA was separated by centrifugation and stored at −80 °C for further use.

### 3.3. Preparation of Pth1:peptidyl-tRNA Complex

Buffers of 20 mM Bis–Tris, 50 mM NaCl and 2 mM DTT were prepared with six different H_2_O:D_2_O percentages, 0, 10%, 18%, 70%, 85% and 100% D_2_O. In separate Slide-A-Lyzer dialysis cassettes (Pierce/Thermo, Rockford, IL, USA), Pth1H20R and peptidyl-tRNA were extensively dialyzed in each of the six buffers. Aliquots of the final dialysis buffer were saved for scattering background subtraction. The concentration of Pth1H20R and bulk peptidyl-tRNA was determined to account for any losses during dialysis before forming a 1:1 complex. The final protein concentration was approximately 2 mg/mL and 2.4 mg/mL peptidyl-tRNA for samples at all D_2_O concentrations.

### 3.4. Dynamic Light Scattering

DLS measurements were performed on a Wyatt DynaPro NanoStar instrument using disposable cuvettes. Pth1H20R and bulk-peptidyl tRNA solutions were prepared as before in H_2_O buffer. Measurements from Pth1H20R, peptidyl-tRNA, and an equal volume mixture (1:1 molar ratio) were collected. The temperature was set to 25 °C and all samples were incubated for 10 min before measurements were initiated.

### 3.5. Small Angle Neutron Scattering of the Pth1:peptidyl-tRNAComplex

Neutron scattering experiments were performed at the High Flux Isotope Reactor at Oak Ridge National Laboratories at beam CG-3, in the cold-guide hall. All samples were 300 μL, added to 1 mm quartz “banjo” cells at room temperature. The sample detector distance was 1.7 meters and 6 Å wavelength neutrons with a wavelength spread, dλ/λ, of 0.15 were used. Exposure times were from 60 min to 240 min, depending on the D_2_O concentration. To compensate for reduced signal to noise, samples with lesser scattering density (*i.e.*, closer to the match point) were run longer. Background scattering for each buffer was also measured, along with empty cuvette, H_2_O, D_2_O, and porasil B standards for data reduction and background subtraction. The calibrated porasil B standard was used to place the scattering data on absolute intensity scale [34]. Data were collected using a phase contrast series with D_2_0 concentrations of 0%, 10%, 18%, 70%, 85% and 100% in the same buffer, allowing for a more complete picture of the complex.

### 3.6. Overall Shape Determination

Data were reduced and analyzed in Igor Pro (WaveMetrics, Lake Oswego, OR, USA) with the SANS macros implemented by Dr. Kenneth Littrell (ORNL) to analyze the overall radius of gyration of the complex using a Guinier approximation [35] before using GNOM [25]. Using the GNOM output as an upper limit for size, low resolution models of the Pth1:peptidyl-tRNA complex were calculated using MONSA [36]. All five data sets at different H_2_O:D_2_O ratios were included. Data were analyzed based on a zero symmetry model. The crystal structure of *E. coli* Pth1 (PDBID:2PTH) [27] was fit in to the shape using SUPCOMB [28].

### 3.7. Chemical Shift Perturbation Mapping of Piperonylpiperazine Binding to Pth1

Chemical shift perturbation mapping was performed for the interaction of wild type *E. coli* Pth1 with piperonylpiperazine, monitoring ^1^H–^15^N backbone resonances from ^15^N-HSQC spectra. Titration data were collected on a Varian Inova 800 MHz spectrometer in an NMR buffer of 20 mM Bis–Tris, 100 mM NaCl, 2 mM TCEP, pH 6.6 at 25 °C. Spectra were recorded for ligand:protein ratios of 0:1, 1:1, 4:1, 16:1, 25:1 and 64:1. A 20 mM stock solution of piperonylpiperazine was titrated into a 250 μL sample of 200 μM ^15^N Pth1. Control spectra were recorded with titration of buffer alone with no differences observable up to the maximum tested volume added.

### 3.8. Computational Docking

*E. coli* Pth1 (PDB ID:2PTH) was used as the receptor for virtual small molecule docking with the ligand piperonylpiperazine using AutoDockVina [37]. Python Molecular Viewer with AutoDock Tools were used for conversion to pdbqt format, required by AutoDockVina [38]. A virtual molecular structure of piperonylpiperazine was generated and the bond angles were optimized using Accelrys Draw, converted to pdb format using Chimera [39], and pdbqt format as for Pth1. Default simulation parameters for smoothing and scoring functions were used for docking simulations. An initial search of the entire protein indicated three possible interaction sites, one agreeing with chemical shift perturbations. Thus the final search space was limited to the region of Pth1 showing chemical shift perturbations in solution NMR studies, with an associated grid box size of 28 × 22 × 20 Å centered at 37.3, 42.9, 69.0 for the *x*, *y*, and *z* centers, respectively. The six lowest energy ligand poses out of 36 calculated were exported as individual PDB files.

## 4. Conclusions

Bacterial Pth1 has been long recognized as a potential target for new antibiotic development. Structure based drug design has been helped by high resolution structures of Pth1 from several pathogenic bacteria. However, the high resolution structural details of complex formation still remain unresolved. There are several issues that make structure determination of the enzyme:substrate complex challenging. First, the production of a homogeneous sample of peptidyl-tRNA in quantities large enough for structural studies has yet to be overcome. Second, the dynamic nature of tRNA is a barrier to crystallization [22]. Here we took advantage of insensitivity of small angle neutron scattering to a heterogeneous sample of peptidyl-tRNA bound to a catalytically inactive H20R mutant of Pth1 to determine the overall shape of the complex. The H20R mutant has been shown to be structurally unperturbed while still binding the substrate [26]. NMR data (not shown) provided evidence that the H20R mutant bound peptidyl-tRNA with high affinity, being completely (>95%) bound at a 1:1 molar ratio.

**Figure 4:**
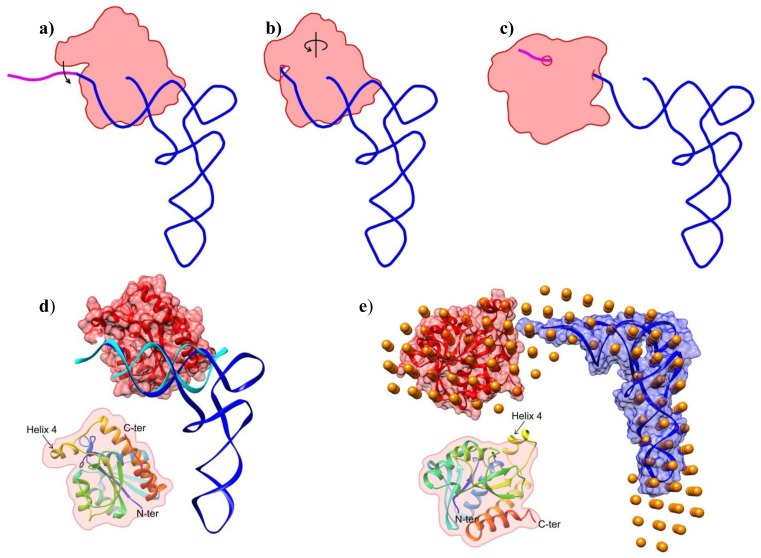
Model of Pth1 Interaction with peptidyl-tRNA. (**a**–**c**) Cartoon representation of the Pth1 (red) interaction model with peptidyl-tRNA (blue and magenta). (**a**) After substrate recognition; (**b**) helix 4 clamps the peptide portion (magenta) and CCA terminus of the substrate in the binding channel; (**c**) followed by the enzymatic reaction and release of products or just release of the nucleotide as observed in the SANS model; (**d**–**e**) Available high and low resolution structures of Pth1 and peptidyl-tRNA on which the model of interaction was built; (**d**) Crystal structures of the complex between Pth1 (PDBID:2PTH, red surface) and the TΨC loop of tRNA (PDBID:3VJR, cyan) with tRNA^Phe^(PDBID:1EHZ, blue) superimposed; (**e**) SANS model (orange beads) of the interaction presented here with the same coloring as in (**d**); Insets show the orientation of Pth1. In black, His20 is the only side chain shown.

The overall shape shows an extended complex with minimal interaction between the tRNA and Pth1. This is somewhat different from the interaction between Pth1 and the TΨC loop of tRNA observed in a high resolution crystal structure, Figure 4d[22]. This may, in part, be due to the presence of an additional base, G-1, in the TΨC structure that was necessary for crystallization. The differences might also be the result of crystallization with the X-ray structure being forced into a low-population state from crystal packing. Also the lack of peptide moiety on the tRNA may be a contributing factor, the ramifications of which are discussed subsequently. In the above model, the CCA terminus appears to be positioned near the catalytic residue 20, a requirement for substrate cleavage. The above model also upholds finding that the D arm, anticodon arm and variable loop do not exist in a location where they interact with Pth1. It appears that while the tight interaction between Pth1 and the TΨC loop of tRNA may be a mode of substrate recognition, the low resolution model of Pth1:peptidyl-tRNA interaction presented here is a later step in the reaction along the lines of product dissociation. From both sets of structural data, we propose the following model of Pth1 interaction with its substrate, Figure 4. In the first step, the enzyme binds tRNA, screening its substrate candidates via the large positively charged patch shown to interact with the tRNA portion of the substrate, as previously proposed [22]. If the nucleotide binding partner has a sufficient peptide component (*i.e.*, more than one amino acid), the peptide binds in the deep cleft next to helix-4, causing it to “close”, clamping the substrate in place. Helix-4 closure, or at least sufficient duration of closure, is necessary for the enzymatic reaction to occur. Once cleaved, helix-4 opens and the reaction products dissociate. In the SANS model presented here, a catalytically inactive Pth1 mutant (that still binds the substrate) was used. Thus the enzymatic reaction did not occur but the tRNA portion of the substrate dissociated from its original binding site. The dissociation might actually serve a functional purpose that is to facilitate accommodation of the peptide in the peptide binding channel without constraints imposed by tRNA binding to Pth1. On the other hand, a considerable strain from bending the acceptor stem to fit the peptide component into the Pth1 peptide recognition channel might aid in cleavage of the tRNA-peptide ester bond. Further studies will be necessary to fully elucidate the intermediate steps.

Finding a small molecule that can bind to Pth1, coupled with natural product extract inhibition [23,24], underscores the utility of Pth1 as a drug target. Though piperonylpiperazine was a common constituent of most compounds with inhibitory activity found in a combinatorial synthetic library, it is not sufficient to inhibit Pth1 by itself. From the above model, piperonylpiperazine binds on the opposite side of Pth1 than the substrate, explaining the lack of inhibition. However, having a small molecule that does bind provides a base from which to build more specific inhibitors. Guided by chemical shift perturbation mapping, computational docking shows favorable interactions with a hydrophobic stretch, leading to the possibility of allosteric regulation.

Though the Pth1:peptidyl-tRNA complex resists high resolution characterization, future studies show promise. SANS data can be incorporated into solution structure refinement by utilizing NOEs to solve the short-range interactions and the SANS data for the shape. This has been particularly helpful for RNA structures [40,41]. Considerable progress has been made with combining tRNA and peptides [42,43], though scale up has been problematic and/or expensive. Continued efforts will help understand the intricate workings of Pth1 enzymes and hopefully fulfill their pharmacological potential.
